# Sex differences in autonomic adverse effects related to antipsychotic treatment and associated hormone profiles

**DOI:** 10.1038/s41537-023-00430-4

**Published:** 2024-01-06

**Authors:** Ingrid T. Johansen, Nils Eiel Steen, Linn Rødevand, Synve H. Lunding, Gabriela Hjell, Monica B. E. G. Ormerod, Ingrid Agartz, Ingrid Melle, Trine V. Lagerberg, Mari Nerhus, Ole A. Andreassen

**Affiliations:** 1 NORMENT Centre, Division of Mental Health and Addiction, Oslo University Hospital & Institute of Clinical Medicine, University of Oslo, Oslo, Norway; 2 Department of Psychiatry, Ostfold Hospital, Graalum, Norway; 3 Department of Psychiatric Research, Diakonhjemmet Hospital, Oslo, Norway; 4 Department of Clinical Neuroscience, Centre for Psychiatric Research, Karolinska Institute & Stockholm Health Care Services, Stockholm Region, Stockholm, Sweden; 5 Division of Health Services Research and Psychiatry, Institute of Clinical Medicine, University of Oslo Campus Ahus, Lørenskog, Norway; 6 Department of Special Psychiatry, Akershus University Hospital, Lørenskog, Norway

**Keywords:** Psychosis, Psychology

## Abstract

Autonomic adverse effects of antipsychotic drugs (APs) cause clinical challenges, but few studies have investigated sex differences and their underlying biological pathways. Sex-specific regulation of relevant hormones could be involved. We investigated sex differences in autonomic adverse effects related to olanzapine, quetiapine, risperidone, and aripiprazole, and the role of hormones related to APs. Patients with severe mental disorders (*N* = 1318) were included and grouped based on AP monotherapy: olanzapine (*N* = 364), quetiapine (*N* = 211), risperidone (*N* = 102), aripiprazole (*N* = 138), and no AP (*N* = 503). Autonomic symptoms from the Udvalg for Kliniske Undersøgelser (UKU) side effect scale was analyzed with logistic regression, adjusting for age, diagnosis, and polypharmacy. Further, we analyzed associations between autonomic symptoms and hormones related to APs. We found associations between autonomic adverse effects and APs, with sex-specific risk for palpitations/tachycardia associated with hormonal changes related to APs. Results showed increased salivation associated with aripiprazole, reduced salivation with quetiapine, and nausea/vomiting and palpitations/tachycardia with olanzapine, and higher risk of nausea/vomiting, diarrhea, constipation, polyuria/polydipsia, and palpitations/tachycardia in females. Significant sex x AP interaction was found for palpitations/tachycardia, with higher risk in risperidone-treated males, which was associated with different hormone profiles of prolactin, cortisol, and insulin. Our findings implicate a role of several hormones in the sex-specific autonomic adverse effects related to APs.

## Introduction

Schizophrenia and bipolar disorders are severe mental disorders (SMD) with overlapping symptomatology and shared susceptibility genes^1–3^. Antipsychotic drugs (APs) are effective for treating several symptoms of SMD^4,5^, but they are also associated with a wide range of negative effects including metabolic disturbance, autonomic, neurological, psychological, sexual, and hormonal adverse effects^6–8^, which affect treatment adherence and quality of life in patients with SMD^9,10^. Variations in receptor binding profiles might explain differences in adverse effects across APs, but the underlying mechanisms are unclear^11^. Sex differences in adverse effects of APs have been indicated^7,12,13^, but sex-specific treatment strategies in patients with SMD are lacking. To improve treatment, we need more knowledge regarding sex-specific adverse effects and explore possible underlying mechanisms.

Dysfunction of the autonomic nervous system (ANS) is linked to increased cardiovascular disease (CVD) risk in patients with SMD^14,15^. Heart rate variability (HRV), an index of cardiac ANS regulation, is reduced in patients with SMD compared to healthy controls^16^. APs affect the ANS, as they act on adrenergic and cholinergic receptors and influence central autonomic regulation and baroreceptor reflexes^15,17^. The Udvalg for Kliniske Undersøgelser (UKU) side effect scale includes the following autonomic adverse effects: accommodation disturbance, increased and reduced salivation, nausea/vomiting, diarrhea, constipation, micturition disturbance, polyuria/polydipsia, orthostatic dizziness, palpitations/tachycardia, and increased tendency of sweating^18^. Although few studies have investigated sex differences in these autonomic adverse effects specifically, there are indications that female patients receiving olanzapine report more autonomic adverse effects than males^19^. In addition, Iversen et al.^8^ found that female patients with SMD had higher risk for accommodation disturbances, nausea, constipation, orthostatic dizziness, and palpitations, but the direct relations to APs were unclear and they did not conduct interaction analyses between sex and APs.

Our research group and others have shown that several hormones are affected by AP treatment, and hormonal changes related to APs might be involved in the underlying mechanisms of sex-specific adverse effects^20^. Hyperprolactinemia has been linked to AP treatment, and females have a higher risk than males^7^. The APs differ in the propensity to elevate prolactin levels, and risperidone seems to have a particularly high risk^21^. Elevated testosterone levels have been found in females with schizophrenia, while reduced levels have been found in males, and the changes in testosterone levels might be linked to the prolactin-releasing effect of APs^22^. Circulating testosterone is bound to sex-hormone binding globulin (SHBG), and lower levels of SHBG have been found in AP-treated females compared to males^23^. Evidence has also shown that APs reduce hypothalamic-pituitary-adrenal (HPA) axis activity and cortisol secretion^24,25^, and a blunted stress response seems to be more evident in males^26^. Also, reduced levels of thyroxine (fT4) and elevated thyroid-stimulating hormone (TSH) levels have been linked to APs, but less is known about potential sex differences^27^. There are some differences across APs in the effect on insulin levels, and females seem more vulnerable to developing insulin resistance compared to males^28,29^. Additionally, different levels of adipokines (i.e., leptin and adiponectin) have been indicated in AP-treated males and females^29^. Despite the indicated link between hormones and APs, and the physiological hormonal differences between males and females, the role of hormones in sex-specific autonomic adverse effects related to APs is unclear. Several hormones and signaling pathways may be involved, and in addition to the hormones described here, estrogen, noradrenaline, adrenaline, and acetylcholine might have a role.

The present study aimed to identify sex differences in autonomic adverse effects related to olanzapine, quetiapine, risperidone, and aripiprazole, and characterize the potential role of hormonal changes related to AP treatment. We first investigated sex differences in autonomic adverse effects related to use of olanzapine, quetiapine, risperidone, and aripiprazole in patients with SMD. For autonomic adverse effects found with a significant interaction effect, we further investigated the associations with hormones related to APs (prolactin, cortisol, TSH, fT4, testosterone, insulin, leptin, adiponectin and SHBG).

## Results

### Demographic and clinical characteristics

Table 1 shows demographic and clinical characteristics of the study sample and for the male and female subsample, including distribution across the AP groups for sex, age, ethnicity, diagnostic group, and current symptom scores. Significant AP group differences were found for all these variables in the total sample, for age, ethnicity, diagnostic groups and PANSS in the male subsample and for age, diagnostic groups, IDS-C and YMRS in the female subsample.

**Table 1:** Demographic and clinical characteristics of the study sample.

	No AP	Olanzapine	Quetiapine	Risperidone	Aripiprazole	*p*
	*N* = 503	*N* = 364	*N* = 211	*N* = 102	*N* = 138	
TOTAL (*N* = 1318)						
Females, *N* (%)	274 (54.5)	141 (38.7)	122 (57.8)	40 (39.2)	64 (46.4)	**<0.001**
Years of age, mean (SD)	32 (11.2)	32 (10.4)	30 (10.4)	31 (10.1)	29 (9.2)	**0.001**
Caucasian, *N* (%)	431 (85.7)	301 (82.7)	181 (85.8)	80 (78.4)	105 (76.1)	**0.002**
Diagnosis, *N* (%)						**<0.001**
Schizophrenia spectrum^a^	107 (21.3)	191 (52.5)	82 (38.9)	71 (69.6)	86 (62.3)	
Other psychosis^b^	71 (14.1)	62 (17.0)	30 (14.2)	13 (12.7)	19 (13.8)	
Bipolar spectrum^c^	301 (59.8)	93 (25.5)	92 (43.6)	12 (11.8)	27 (19.6)	
Major depressive disorder	24 (4.8)	17 (4.7)	7 (3.3)	6 (5.9)	5 (3.6)	
Total symptom scores, mean (SD)						
PANSS	52.0 (15.6)	55.8 (15.8)	53.9 (14.9)	60.9 (17.0)	55.8 (15.6)	**<0.001**
CDSS	5.6 (4.4)	4.6 (4.3)	5.6 (4.7)	5.6 (4.6)	5.0 (5.0)	**0.014**
IDS-C	18.7 (12.0)	15.1 (11.4)	18.4 (12.0)	18.7 (12.7)	16.0 (12.1)	**<0.001**
YMRS	4.1 (4.5)	3.7 (4.5)	4.2 (5.1)	4.6 (4.8)	3.7 (5.0)	**0.049**
AP dose (mg), mean (SD)	–	12.1 (6.9)	385.9 (321.4)	3.3 (1.5)	13.2 (6.3)	
MALES (*N* = 677)						
Years of age, mean (SD)	33 (11.5)	31 (9.5)	30 (10.1)	31 (9.2)	28 (8.2)	**0.040**
Caucasian, *N* (%)	198 (86.5)	175 (78.5)	72 (80.9)	49 (79.0)	51 (68.9)	**0.001**
Diagnosis, *N* (%)						**<0.001**
Schizophrenia spectrum^a^	60 (26.2)	119 (53.4)	43 (48.3)	46 (74.2)	52 (70.3)	
Other psychosis^b^	43 (18.8)	43 (19.3)	17 (19.1)	9 (14.5)	8 (10.8)	
Bipolar spectrum^c^	113 (49.3)	50 (22.4)	27 (30.3)	4 (6.5)	11 (14.9)	
Major depressive disorder	13 (5.7)	11 (4.9)	2 (2.2)	3 (4.8)	2 (2.7)	
Total symptom scores, mean (SD)						
PANSS	54.6 (16.7)	57.9 (15.2)	57.7 (14.6)	63.2 (15.6)	60.2 (16.8)	**<0.001**
CDSS	5.0 (4.1)	4.5 (4.3)	5.0 (3.9)	5.3 (4.4)	5.1 (5.0)	0.421
IDS-C	17.2 (12.1)	15.0 (11.4)	18.6 (10.9)	18.2 (12.4)	16.7 (12.2)	0.101
YMRS	4.1 (4.5)	4.0 (4.7)	4.6 (5.4)	4.7 (4.6)	3.7 (4.7)	0.686
AP dose (mg), mean (SD)	–	13.1 (7.7)	435.3 (373.3)	3.5 (1.6)	13.5 (5.3)	
FEMALES (*N* = 641)						
Years of age, mean (SD)	32 (10.9)	35 (11.1)	30 (10.6)	32 (11.4)	30 (10.2)	**<0.001**
Caucasian, *N* (%)	233 (85.0)	126 (89.4)	109 (89.3)	31 (77.5)	54 (84.4)	0.379
Diagnosis, *N* (%)						**<0.001**
Schizophrenia spectrum^a^	47 (17.2)	72 (51.1)	39 (32.0)	25 (62.5)	34 (53.1)	
Other psychosis^b^	28 (10.2)	19 (13.5)	13 (10.7)	4 (10.0)	11 (17.2)	
Bipolar spectrum^c^	188 (68.6)	43 (30.5)	65 (53.3)	8 (20.0)	16 (25.0)	
Major depressive disorder	11 (4.0)	6 (4.3)	5 (4.1)	3 (7.5)	3 (4.1)	
Total symptom scores, mean (SD)						
PANSS	49.9 (14.3)	52.4 (16.2)	51.0 (14.5)	57.4 (18.6)	50.9 (12.5)	0.103
CDSS	6.2 (4.6)	4.9 (4.3)	6.0 (5.2)	5.9 (5.0)	4.9 (5.1)	0.066
IDS-C	19.7 (11.8)	15.3 (11.4)	18.3 (12.7)	19.4 (13.3)	15.1 (12.0)	**0.007**
YMRS	4.2 (4.5)	3.1 (4.1)	4.0 (4.9)	4.5 (5.1)	3.6 (5.3)	**0.020**
AP dose (mg), mean (SD)	–	10.4 (5.0)	350.2 (273.9)	2.9 (1.4)	12.8 (7.2)	

Table 1 shows the demographical and clinical characteristics of patients with SMD, in the total sample and for the male/female subsample. Differences across AP groups were investigated using Kruskal-Wallis test for age and symptom scores, and chi-square test for diagnostic groups and ethnicity. The corresponding *p*-values are shown in the table with significant results marked in bold.

*AP* Antipsychotics, *PANSS* Positive and Negative Symptom Score, *CDSS* Calgary depression scale for Schizophrenia, *IDS-C* Inventory of depressive symptomatology clinical rating, *YMRS* Young Mania Rating Scale, *SD* Standard deviation.

^a^Schizophrenia spectrum = schizophrenia, schizophreniform disorder, schizoaffective disorder.

^b^Other psychosis = brief psychotic disorder, delusional disorder, psychosis not otherwise specified (NOS).

^c^Bipolar spectrum = bipolar disorder I, bipolar disorder II, bipolar NOS.

### Sex differences in autonomic adverse effects related to antipsychotic treatment

**Fig. 1:**
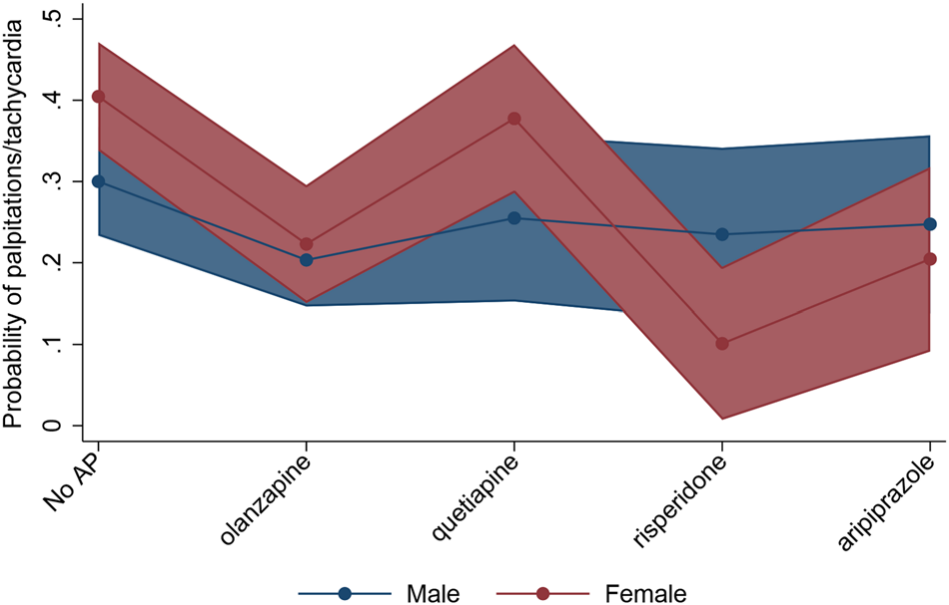
Interaction plot showing the probability of palpitations/tachycardia across antipsychotic (AP) groups in male and female patients with severe mental disorders. The figure illustrates the significant interaction effect for palpitations/tachycardia (*p* = 0.021), with the probability and 95% confidence interval for males and females. Palpitations/tachycardia (yes/no) was the dependent variable in the logistic regression analysis, adjusting for age, diagnostic groups, and non-AP psychopharmacological drugs. Risperidone-treated males show a higher probability for palpitations/tachycardia compared to risperidone-treated females.

The results from the logistic regression analyses investigating sex differences in autonomic adverse effects related to AP groups are presented in Table 2. Significant main effect for AP groups were found for increased salivation (No AP vs. aripiprazole, *p* = 0.018), reduced salivation (No AP vs. quetiapine, *p* = 0.003), nausea/vomiting (No AP vs. olanzapine, *p* = 0.023) and palpitations/tachycardia (No AP vs. olanzapine, *p* = 0.035). Significant main effect for sex was found for nausea/vomiting (*p* = 0.007), diarrhea (*p* = 0.025), constipation (*p* = 0.043), polyuria/polydipsia (*p* = 0.008) and palpitations/tachycardia (*p* = 0.028), all with higher risk in females. A significant interaction effect between sex and AP groups ([males;females x No AP;risperidone] *p* = 0.021) was found for palpitations/tachycardia, with higher risk in males treated with risperidone (Fig. 1).

**Table 2:** Results from the binary logistic regression analyses of autonomic adverse effects.

Dependent variable	Main effect									
	No AP vs. olanzapine		No AP vs. quetiapine		No AP vs. risperidone		No AP vs. aripiprazole		Sex	
	*OR (95% CI)*	*p*	*OR (95% CI)*	*p*	*OR (95% CI)*	*p*	*OR (95% CI)*	*p*	*OR (95% CI)*	*p*
Accommodation disturbance	0.942 (0.474–1.873)	0.865	1.035 (0.432–2.480)	0.938	1.291 (0.512–3.252)	0.588	1.231 (0.474–3.197)	0.670	1.121 (0.605–2.074)	0.717
Increased salivation	0.989 (0.389–2.516)	0.982	1.091 (0.325–3.661)	0.888	2.282 (0.742–7.013)	0.150	3.402 (1.229–9.413)	**0.018**	0.370 (0.123–1.118)	0.078
Reduced salivation	1.246 (0.775–2.004)	0.364	2.495 (1.365–4.561)	**0.003**	1.574 (0.794–3.119)	0.194	0.598 (0.265–1.349)	0.216	1.145 (0.737–1.779)	0.546
Nausea/vomiting	0.504 (0.279–0.912)	**0.023**	0.896 (0.442–1.819)	0.762	0.699 (0.304–1.607)	0.400	0.604 (0.255–1.426)	0.250	1.885 (1.187–2.995)	**0.007**
Diarrhea	0.835 (0.449–1.554)	0.570	1.787 (0.872–3.659)	0.113	0.511 (0.166–1.579)	0.244	1.133 (0.468–2.743)	0.782	1.800 (1.078–3.007)	**0.025**
Constipation	1.022 (0.513–2.036)	0.950	1.040 (0.423–2.560)	0.932	0.984 (0.359–2.698)	0.975	0.981 (0.361–2.669)	0.970	1.834 (1.019–3.301)	**0.043**
Micturition disturbance	0.829 (0.328–2.096)	0.692	1.524 (0.539–4.312)	0.427	2.274 (0.794–6.513)	0.126	0.843 (0.214–3.310)	0.806	0.588 (0.233–1.486)	0.262
Polyuria/Polydipsia	1.367 (0.770–2.427)	0.285	1.158 (0.536–2.502)	0.708	0.916 (0.364–2.309)	0.853	1.562 (0.682–3.575)	0.291	1.994 (1.196–3.325)	**0.008**
Orthostatic dizziness	0.609 (0.367–1.008)	0.054	0.656 (0.333–1.295)	0.224	0.793 (0.390–1.611)	0.521	1.186 (0.609–2.309)	0.617	1.448 (0.937–2.236)	0.096
Palpitations/Tachycardia	0.583 (0.353–0.963)	**0.035**	0.790 (0.410–1.521)	0.481	0.706 (0.347–1.436)	0.337	0.758 (0.375–1.534)	0.441	1.623 (1.055–2.498)	**0.028**
Increased tendency of sweating	0.587 (0.321–1.071)	0.083	0.928 (0.448–1.924)	0.842	0.586 (0.223–1.539)	0.279	1.363 (0.614–3.027)	0.446	1.339 (0.829–2.159)	0.232

	Interaction effect									
	Sex x No AP vs. olanzapine		Sex x No AP vs. quetiapine		Sex x No AP vs. risperidone		Sex x No AP vs. aripiprazole			
	*OR (95% CI)*	*p*	*OR (95% CI)*	*p*	*OR (95% CI)*	*p*	*OR (95% CI)*	*p*		
Accommodation disturbance	1.604 (0.642–4.007)	0.312	1.217 (0.409–3.628)	0.724	0.950 (0.240–3.753)	0.941	2.226 (0.660–7.509)	0.197		
Increased salivation	3.280 (0.774–14.762)	0.105	3.062 (0.571–16.404)	0.191	1.933 (0.306–12.224)	0.484	1.972 (0.406–9.594)	0.400		
Reduced salivation	0.996 (0.513–1.933)	0.990	1.167 (0.541–2.515)	0.694	0.827 (0.302–2.267)	0.712	2.301 (0.808–6.558)	0.119		
Nausea/vomiting	0.538 (0.229–1.267)	0.156	0.753 (0.316–1.798)	0.523	1.065 (0.335–3.386)	0.916	1.159 (0.382–3.521)	0.795		
Diarrhea	0.532 (0.223–1.271)	0.155	0.428 (0.172–1.065)	0.068	0.890 (0.186–4.252)	0.884	0.700 (0.215–2.278)	0.554		
Constipation	0.742 (0.298–1.842)	0.520	1.871 (0.663–5.282)	0.237	0.726 (0.176–2.994)	0.658	0.667 (0.172–2.584)	0.558		
Micturition disturbance	1.166 (0.272–4.990)	0.836	0.634 (0.130–3.092)	0.573	0.311 (0.029–3.227)	0.328	2.744 (0.437–17.246)	0.282		
Polyuria/Polydipsia	0.498 (0.228–1.089)	0.081	0.729 (0.285–1.863)	0.509	1.000 (0.195–1.774)	1.000	0.5886 (0.195–1.774)	0.346		
Orthostatic dizziness	1.160 (0.579–2.324)	0.674	1.789 (0.782–4.095)	0.168	2.347 (0.859–6.411)	0.096	0.806 (0.318–2.046)	0.650		
Palpitations/Tachycardia	0.695 (0.342–1.415)	0.316	1.122 (0.498–2.529)	0.781	0.219 (0.061–0.796)	**0.021**	0.478 (0.170–1.341)	0.161		
Increased tendency of sweating	1.141 (0.506–2.570)	0.751	1.044 (0.425–2.564)	0.925	0.920 (0.229–3.691)	0.907	0.299 (0.085–1.054)	0.060		

Table 2 shows the results from the binary logistic regression models investigating sex differences in autonomic adverse effects related to APs. The main effects of the AP groups and interaction effects between sex and AP groups are shown (from the same logistic regression model), with significant results marked in bold.

The No AP group was the reference group for the AP group variable, and males for the sex variable.

*AP* Antipsychotics, *OR* Odds ratio, *CI* Confidence interval, *vs*. versus.

### Associations with hormones related to antipsychotic treatment

Table 3 shows the results from the logistic regression analyses including hormones related to APs in the statistical model with palpitations/tachycardia as dependent variable. The significant interaction effect found for palpitations/tachycardia became non-significant in all models including a hormone, suggesting that the sex difference found for palpitations/tachycardia is associated with different hormone levels. Of the hormones investigated, prolactin (*p* = 0.007), cortisol (*p* = 0.028), and insulin (*p* = 0.035) showed a significant main effect, indicating a specific role of these hormones.

**Table 3:** Relationship between sex differences in palpitations/tachycardia related to risperidone and antipsychotic related hormones.

Hormone (independent variable)	Main effect: hormone		Interaction effect: Sex x No AP vs. risperidone	
	*OR (95% CI)*	*p*	*OR (95% CI)*	*p*
Prolactin, mU/L	1.001 (1.000–1.001)	**0.007**	0.227 (0.043–1.202)	0.081
Testosterone, nmol/L	1.029 (0.988–1.072)	0.171	0.327 (0.067–1.594)	0.166
SHBG, nmol/L	0.997 (0.992–1.003)	0.322	0.328 (0.067–1.601)	0.168
Leptin, pmol/L	1.000 (0.999–1.000)	0.276	0.361 (0.075–1.749)	0.206
Adiponectin, mg/L	0.995 (0.967–1.025)	0.757	0.388 (0.079–1.883)	0.240
Insulin, pmol/L	1.002 (1.000–1.004)	**0.035**	0.402 (0.083–1.951)	0.258
Cortisol, nmol/L	1.001 (1.000–1.002)	**0.028**	0.376 (0.078–1.827)	0.225
TSH, mIE/L	0.954 (0.846–1.075)	0.435	0.370 (0.094–1.455)	0.155
fT4, pmol/L	1.057 (0.997–1.121)	0.061	0.347 (0.088–1.374)	0.132

Table 3 shows the results for the logistic regression models investigating the role of hormones in associations with the sex difference found for palpitations/tachycardia. Palpitations/tachycardia was the dependent variable, and each hormone was added to the model one at the time, adjusted for age, diagnostic group, and other psychopharmacological drugs. We investigated potential change in significance of the interaction effect between sex and No AP vs. risperidone which was found significant in the first step (*p* = 0.021), and the main effect of each hormone. Significant *p*-values are marked in bold.

*AP* Antipsychotics, *SHBG* Sex-hormone binding globulin, *TSH* Thyroid-stimulating hormone, *fT4* free thyroxine, *OR* Odds ratio, *CI* Confidence interval, *vs*. versus.

### Sensitivity analyses

The results from the sensitivity analyses adjusting for AP compliance showed similar results as in the full sample, except for palpitations/tachycardia where the result for No AP vs. olanzapine did not reach significance (*p* = 0.051). The results from the sensitivity analyses are described in detail in the Supplementary material.

## Discussion

The main findings of the present study were associations between autonomic adverse effects and individual APs, and a significant interaction between sex and AP groups for palpitations/tachycardia, with higher risk in risperidone-treated males. This sex-specific finding was associated with hormone levels suspected to be influenced by APs, and sex-dependent regulation of hormones related to AP treatment thus seem to have a role in the sex-specific autonomic adverse effects.

We found associations between individual APs and several autonomic adverse effects independently of sex. Patients with SMD receiving aripiprazole had a higher risk of increased salivation, and quetiapine-treated patients had a higher risk of reduced salivation compared to patients not receiving any APs. Olanzapine-treated patients had lower risk of nausea/vomiting and palpitations/tachycardia compared to no AP users. APs have variations in receptor binding profile, and compared to olanzapine and quetiapine, risperidone and aripiprazole have shown a lower affinity to the muscarinic receptors, indicating that olanzapine and quetiapine reduce the ANS activity to a greater extent compared to risperidone and aripiprazole^11,17^. This is in accordance with our findings, and variations between individual APs in the effect on ANS might partly explain differences in AP adverse effects.

We show differences between males and females for nausea/vomiting, diarrhea, constipation, polyuria/polydipsia, and palpitations/tachycardia, with a higher risk in female patients with SMD. These results show the main effect of sex and were not linked to APs per se. Therefore, these results indicate that differences between males and females can be due to causes also different from AP treatment. In general, females have greater parasympathetic responsiveness and males have greater sympathetic responsiveness^30^. It might be that differences in the effect of ANS between males and females cause differences in autonomic symptoms, regardless of AP treatment. Iversen et al.^8^ showed in a partly overlapping sample that female patients with SMD had a higher risk of accommodation disturbance, nausea, constipation, orthostatic dizziness, and palpitations/tachycardia, which our findings are in accordance with. However, few studies have investigated sex-specific effects of ANS in patients with SMD independently of AP treatment, and more knowledge is needed to better understand these associations.

Studies investigating sex-specific autonomic adverse effects related to individual APs are few. Still, we report sex-specific findings related to AP treatment for palpitations/tachycardia, with a higher risk in males receiving risperidone. Howell et al.^14^ showed that risperidone was associated with a moderate risk of palpitations, but they did not conduct sex-specific analyses. In addition to differences in the affinity to muscarinic receptors across APs, it is also shown that risperidone has a higher alpha1-adrenoceptor antagonist activity, which causes a greater sympathetic discharge^15^. This might partly explain the differences seen for palpitations/tachycardia, but it is also reasonable to think that the mechanisms are more complex. Sex hormones such as estrogen and testosterone seem to be involved in the differences in ANS function between males and females^30^, but our findings suggest that also sex-specific hormonal changes related to APs have a role. It might be that these hormones act through ANS pathways, as there is evidence of sex differences in functioning of the ANS^30^. Our results showed the potential role of several hormonal changes related to AP treatment, specifically with prolactin, cortisol, and insulin. Risperidone is one of the APs with the highest risk of prolactin elevation and females are more susceptible to this adverse effect^7,21,28^. This in accordance with our findings of sex-dependent prolactin levels. Further, our results suggest that sex-dependent prolactin levels could be involved in autonomic adverse effects. However, the underlying mechanisms of sex-specific AP adverse effects need clarification in experimental studies.

Our findings are important for clinicians treating patients with SMD, who should be aware of sex-specific differences in the experience of palpitations/tachycardia related to AP treatment. Autonomic adverse effects have previously been associated with poor medication adherence in patients with SMD^31^. Palpitations cause discomfort and affect the quality of life, and it is important to monitor the heart rate in patients receiving APs, and based on our results, in risperidone-treated male patients especially. Clinicians should also be aware of the associations between several autonomic adverse effects and individual APs, as some seem to be AP specific, but with similar risks for males and females. It is also important that female patients with SMD seem to have a higher risk of certain autonomic symptoms regardless of AP treatment, and clinicians should examine, monitor, and treat autonomic symptoms also independently of AP use. ANS has a major role in the regulation of the cardiovascular system under both psychological and pathophysiological conditions^32^, and our findings might be further linked to the increased risk of cardiovascular disease (CVD) in patients with SMD^33,34^, in which the underlying mechanisms remain unknown. Altogether, the underlying mechanisms of AP adverse effects are multifactorial and complex, but our results provide findings for understanding sex-dependent mechanisms which are important for future sex-specific treatment strategies and personalized treatment in patients with SMD. However, before implications are implemented in clinical guidelines, our findings should be tested in experimental studies.

The strengths of the present study include a large well-described sample. We have analyzed sex differences using interaction analyses with patients not using any APs as comparison and we adjusted for several confounders including polypharmacy (antidepressants, mood stabilizers). We used a transdiagnostic sample and did not study the specific diagnoses separately. However, to minimize the potential effect of specific diagnoses, the diagnostic groups were adjusted for in the statistical analyses, and we used patients not using APs as a comparison group. Also, a comprehensive set of hormones related to AP treatment were included. The AP drug groups were based on the mostly used APs in our study sample, which reflect the prescription practice at psychiatric hospitals in Norway. The items from the UKU side effect scale are reported as subjective symptoms and represent distress/discomfort that the patients experience in daily life, which also embodies quality of life. The subjective measures might have been influenced by individual perceptions and communication styles, and by sex differences in the communication of adverse effects^12^. This potential bias may have affected the data of adverse effect^35^. However, this has not affected our objective measures of hormone levels. The well-characterized study sample enabled us to adjust for several confounders, but the autonomic symptoms can be influenced by multiple factors, and there may be other potential confounding factors such as lifestyle and dietary habits that was not considered. The association reported between aripiprazole and increased salivation should be interpreted with caution due to the small number of patients reporting increased salivation. Due to the cross-sectional study design, we cannot conclude regarding causality, and our findings should be replicated in experimental and longitudinal studies to clarify the causal effects. Another limitation of the present study is the lack of information about estrogen levels, which may have impact on AP treatment, and about levels of noradrenaline, adrenaline, and acetylcholine, which are neurotransmitters involved in the ANS. As the SMD patients were predominantly young adults, the findings may be less representative for older age groups, especially postmenopausal females, as the number of postmenopausal females in the sample was low (age ≥52, *N* = 43). Patients were instructed to meet for blood sampling fasting, but information about fasting status was only recorded in a subsample, in which 87% were confirmed fasting. A potential sex difference in fasting status was investigated, with non-significant results. Thus, fasting status is unlikely to be a confounder in the main statistical analyses.

## Conclusions

We found associations between several autonomic adverse effects and AP treatment. Compared to patients with SMD not using any AP, aripiprazole-treated patients had a higher risk of increased salivation, quetiapine-treated showed a higher risk of reduced salivation, and olanzapine-treated had lower risk of nausea/vomiting and palpitations/tachycardia. Sex differences were found for nausea/vomiting, diarrhea, constipation, polyuria/polydipsia, and palpitations/tachycardia, but significant interaction between sex and AP groups was found for palpitations/tachycardia only, with higher risk in risperidone-treated males. Hormonal changes related to APs might have a role in the sex-specific findings for palpitations/tachycardia related to risperidone. These findings underscore the importance of sex-dependent factors in adverse effects related to AP treatment.

## Methods

### Study sample

The study was part of the ongoing Thematically Organized Psychosis (TOP) study (http://www.med.uio.no/norment/english/), recruiting participants from the catchment area of psychiatric units at the major hospitals in Oslo, Norway. The present study was cross-sectional, using data from *N* = 1318 patients with SMD (48.6% (*N* = 641) females). All patients were between 18 and 65 years of age and able to give written informed consent.

Patients with SMD included schizophrenia spectrum disorders (*N* = 537) [schizophrenia, schizophreniform disorder, schizoaffective disorder], other psychotic disorders (*N* = 195) [delusional disorder, brief psychotic disorder, psychotic disorder not otherwise specified (NOS)], bipolar spectrum disorders (*N* = 525) [bipolar I disorder, bipolar II disorder, bipolar disorder NOS] or major depressive disorder with psychotic symptoms (*N* = 59). Patients with a pronounced cognitive deficit, severe somatic disease, history of severe head trauma, or not speaking a Scandinavian language were excluded from the study.

### Clinical assessment

All patients with SMD went through a comprehensive clinical assessment. Diagnosis was established with the Structured Clinical Interview (SCID-I)^36^ for the Diagnostic and Statistical Manual of Mental Disorders, fourth edition (DSM-IV)^37^. Current symptoms were evaluated with Positive and Negative Syndrome Scale (PANSS)^38^, Young Mania Rating Scale (YMRS)^39^, Calgary Depression Scale for Schizophrenia (CDSS)^40^ and Inventory of Depressive Symptomatology Clinician (IDS-C)^41^.

### The UKU side effect rating scale

We used the Udvalg for Kliniske Undersøkelser (UKU) side effect rating scale^18^ to assess adverse effects. This scale was developed and validated for use in psychiatric patients to assess the adverse effects of psychopharmacological drugs and is used to measure the type and severity of adverse effects. It is designed as a semi-structured interview to be performed by trained investigators. The UKU items are divided into four major domains: psychiatric, neurological, autonomic, and other adverse effects, with 48 items in total. All items are scored from 0 to 3 where 0 indicates no symptom present and scores 1 to 3 indicate the presence of a symptom with increasing severity. The present study included items from the autonomic domain: accommodation disturbance, increased salivation, reduced salivation, nausea/vomiting, diarrhea, constipation, micturition disturbance, polyuria/polydipsia, orthostatic dizziness, palpitations/tachycardia, and increased tendency of sweating. These items were reported as subjective symptoms with or without presence the past 7 days and assessed without consideration of causality. The distribution of autonomic adverse effects across AP groups is shown in Supplementary Table 1.

### Psychopharmacological drugs

We collected information about use of psychopharmacological drugs as part of the clinical assessment, and medical records were used to ensure information quality. To obtain standardized doses for mood stabilizers (lithium, antiepileptic drugs) and antidepressants, we applied Defined Daily Dose (DDD) (http://www.whocc.no/atc_ddd_index/). Serum concentrations of APs were used for assessment of AP compliance and analyzed at the Department of Clinical Pharmacology, St. Olav University Hospital, Trondheim, Norway. We included patients independent of their AP dose and the AP doses are shown in Table 1.

### Antipsychotic drug groups

Patients were grouped based on the most frequently used APs, and patients using several APs simultaneously were excluded from the present study. Thus, the groups comprised patients using the following drug as their only AP:

1. *Olanzapine* (*N* = 364, 38.7% (*N* = 141) females). In this group, 7.9% (*N* = 29) received lithium, 17.0% (*N* = 63) antiepileptic drugs, and 32.1% (*N* = 117) antidepressants.
2. *Quetiapine* (*N* = 211, 57.8% (*N* = 122) females). In this group, 11.4% (*N* = 24) received lithium, 27.5% (*N* = 58) antiepileptic drugs, and 42.7% (*N* = 90) antidepressants.
3. *Risperidone* (*N* = 102, 39.2% (*N* = 40) females). In this group, 3.9% (*N* = 4) received lithium, 14.7% (*N* = 15) antiepileptic drugs, and 39.2% (*N* = 40) antidepressants.
4. *Aripiprazole* (*N* = 138, 46.4% (*N* = 64) females). In this group, 5.1% (*N* = 7) received lithium, 12.3% (*N* = 17) antiepileptic drugs, and 25.4% (*N* = 35) antidepressants.
5. *No AP* (*N* = 503, 54.5% (*N* = 274) females). Patients reporting no use of AP. In this group, 8.5% (*N* = 43) received lithium, 26.4% (*N* = 133) antiepileptic drugs, and 31.4% (*N* = 158) antidepressants.

### Psychopharmacological drugs other than antipsychotics

A total of 8.1% (*N* = 107) reported use of lithium, 21.7% (*N* = 286) antiepileptic drugs and 33.4% (*N* = 440) use of antidepressants.

### Hormones related to antipsychotic treatment

Serum levels of prolactin (*N* = 903), cortisol (*N* = 902), thyroid-stimulating hormone (TSH, *N* = 1110), free thyroxine (fT4, *N* = 1105), testosterone (*N* = 918), insulin (*N* = 914), leptin (*N* = 919), adiponectin (*N* = 911), and sex-hormone binding globulin (SHBG, *N* = 899) were assessed. Hormone levels across AP groups are shown in Supplementary Table 2. Thyroid hormones were analyzed at the Department of Medical Biochemistry, Oslo University Hospital, Norway. All other hormones were measured at the Hormone Laboratory, Oslo University Hospital, Norway. The analyses were performed on routine instruments with standard methods. The methods were accredited according to NS-EN ISO/IEC 17025:2017. Methodological changes during the period and adjustments for these are described in detail in the Supplementary material.

### Somatic medications

We collected information regarding current use of somatic medications, and 0.4% (*N* = 5) patients reported use of beta blocker, 3.2% (*N* = 42) levothyroxine, 0.6% (*N* = 8) insulin, 0.08% (*N* = 1) testosterone and 3.9% (*N* = 51) contraceptives.

### Statistical analyses

All statistical analyses were performed using Stata/MP statistical software, version 16. The significance level for the statistical tests was pre-set at *p* ≤ 0.05 (two-tailed). Differences across AP groups in demographic and clinical characteristics were investigated using Kruskal-Wallis test for continuous variables, and chi-square test for categorical variables.

Binary logistic regression analysis was used as the main statistical method. We followed the study hypotheses strictly and systematically, and due to hypothesis-driven analysis design we did not adjust for multiple testing. We summarized scoring 1–3 for each autonomic item from the UKU side effect scale as “present” and used the dichotomized scores: item present (score 1–3) or not (score 0). Each autonomic item was used as a dependent variable one at a time: accommodation disturbance, increased salivation, reduced salivation, nausea/vomiting, diarrhea, constipation, micturition disturbance, polyuria/polydipsia, orthostatic dizziness, palpitations/tachycardia, and increased tendency of sweating. Sex and AP groups were used as independent variables, investigating both main effects and interaction effects between sex and AP groups in the same logistic regression model. We adjusted for age, diagnostic group, and other psychopharmacological drugs, including individual DDD of lithium, antiepileptic drugs, and antidepressants. The No AP group was the reference group for the AP group variable, and males were the reference group for the sex variable.

For autonomic adverse effects found with significant interaction effect between sex and AP groups, we investigated the associations with hormones related to APs (prolactin, cortisol, TSH, fT4, testosterone, insulin, leptin, adiponectin, SHBG). One hormone was added to the logistic regression model at the time. Potential changes in the significance of the interaction effect between sex and AP groups were investigated, as well as the main effect of each hormone.

We did sensitivity analyses to adjust for the potential confounding effect of AP compliance. The statistical analyses were reran in a subsample of patients removing individuals with unsatisfactory compliance (<75%, *N* = 40), using existing criteria^42^.

### Supplementary information

Supplementary Material

## Data Availability

The dataset used in the present study is not publicly available due to ethical restrictions but can be made available from the corresponding author upon reasonable request and approval from the Regional Ethics Committee.
