# Supplementary material for: Sex differences in autonomic adverse effects related to antipsychotic treatment and associated hormone profiles
Source: Schizophrenia (Heidelb). 2024 Jan 6;10(1):6. doi: 10.1038/s41537-023-00430-4 (PMC10851697; doi:10.1038/s41537-023-00430-4)
Supplement: Supplementary file 1 — Supplementary Material [file 41537_2023_430_MOESM1_ESM.docx]

**Supplementary information**

**Supplementary Table 1:** Distribution of autonomic adverse effects across antipsychotic (AP) groups, in the total sample and the male/female subsample

|  | **No AP** | **Olanzapine** | **Quetiapine** | **Risperidone** | **Aripiprazole** |
| --- | --- | --- | --- | --- | --- |
| *Total, N* | 503 | 364 | 211 | 102 | 138 |
| *Females, N (%)* | 274 (54.5) | 141 (38.7) | 122 (57.8) | 40 (39.2) | 64 (46.4) |
| ***UKU item present, N (%)*** |  |  |  |  |  |
| **TOTAL SAMPLE (N=1318)** |  |  |  |  |  |
| *Accommodation disturbance* | 50 (9.9) | 41 (11.3) | 25 (11.8) | 13 (12.7) | 19 (13.8) |
| *Increased salivation* | 15 (3.0) | 18 (4.9) | 11 (5.2) | 9 (8.8) | 15 (10.9) |
| *Reduced salivation* | 129 (25.6) | 105 (28.8) | 94 (44.5) | 33 (32.4) | 26 (18.8) |
| *Nausea/vomiting* | 115 (22.9) | 36 (9.9) | 42 (19.9) | 18 (17.6) | 20 (14.5) |
| *Diarrhoea* | 88 (17.5) | 39 (10.7) | 37 (17.5) | 8 (7.8) | 17 (12.3) |
| *Constipation* | 63 (12.5) | 38 (10.4) | 42 (19.9) | 11 (10.8) | 12 (8.7) |
| *Micturition disturbance* | 20 (4.0) | 15 (4.1) | 11 (5.2) | 9 (8.8) | 7 (5.1) |
| *Polyuria/Polydipsia* | 90 (17.9) | 59 (16.2) | 36 (17.1) | 15 (14.7) | 21 (15.2) |
| *Orthostatic dizziness* | 135 (26.8) | 74 (20.3) | 59 (28.0) | 34 (33.3) | 37 (26.8) |
| *Palpitations/Tachycardia* | 143 (28.4) | 68 (18.7) | 56 (26.5) | 19 (18.6) | 25 (18.1) |
| *Increased tendency of sweating* | 100 (19.9) | 44 (12.1) | 41(19.4) | 11 (10.8) | 16 (11.6) |
| **MALES (N=677)** |  |  |  |  |  |
| *Accommodation disturbance* | 21 (9.2) | 19 (8.5) | 9 (10.1) | 8 (12.9) | 7 (9.5) |
| *Increased salivation* | 10 (4.4) | 10 (4.5) | 4 (4.5) | 6 (9.7) | 9 (12.2) |
| *Reduced salivation* | 53 (23.1) | 58 (26.0) | 34 (38.2) | 20 (22.5) | 9 (12.2) |
| *Nausea/vomiting* | 37 (16.2) | 22 (9.9) | 14 (15.7) | 9 (10.1) | 8 (10.8) |
| *Diarrhoea* | 29 (12.7) | 23 (10.3) | 16 (17.9) | 4 (4.5) | 8 (10.8) |
| *Constipation* | 19 (8.3) | 20 (8.9) | 8 (8.9) | 6 (9.7) | 6 (8.1) |
| *Micturition disturbance* | 11 (4.8) | 10 (4.5) | 7 (7.9) | 8 (12.9) | 3 (4.1) |
| *Polyuria/Polydipsia* | 28 (12.2) | 34 (15.2) | 12 (13.5) | 7 (11.3) | 10 (13.5) |
| *Orthostatic dizziness* | 51 (22.2) | 38 (17.0) | 15 (16.9) | 15 (24.2) | 20 (27.0) |
| *Palpitations/Tachycardia* | 53 (23.1) | 39 (17.5) | 17 (19.1) | 15 (24.2) | 15 (20.2) |
| *Increased tendency of sweating* | 38 (16.6) | 21 (9.4) | 14 (15.7) | 6 (9.7) | 11 (14.9) |
| **FEMALES (N=641)** |  |  |  |  |  |
| *Accommodation disturbance* | 29 (10.6) | 22 (15.6) | 16 (13.1) | 5 (12.5) | 12 (18.8) |
| *Increased salivation* | 5 (1.8) | 8 (5.7) | 7 (5.7) | 3 (7.5) | 6 (9.4) |
| *Reduced salivation* | 76 (27.7) | 47 (33.3) | 60 (49.2) | 13 (32.5) | 17 (26.6) |
| *Nausea/vomiting* | 78 (28.5) | 14 (9.9) | 28 (22.9) | 9 (22.5) | 12 (18.8) |
| *Diarrhoea* | 59 (21.5) | 16 (11.3) | 21 (17.2) | 4 (10.0) | 9 (14.1) |
| *Constipation* | 44 (16.1) | 18 (12.8) | 34 (27.9) | 5 (12.5) | 6 (9.4) |
| *Micturition disturbance* | 9 (3.3) | 5 (3.5) | 4 (3.3) | 1 (2.5) | 4 (6.3) |
| *Polyuria/Polydipsia* | 62 (22.6) | 25 (17.7) | 24 (19.7) | 8 (20.0) | 11 (17.2) |
| *Orthostatic dizziness* | 84 (30.7) | 36 (25.5) | 44 (36.1) | 19 (47.5) | 17 (26.6) |
| *Palpitations/Tachycardia* | 90 (32.8) | 29 (20.6) | 39 (31.9) | 4 (10.0) | 10 (15.6) |
| *Increased tendency of sweating* | 62 (22.6) | 23 (16.3) | 27 (22.1) | 5 (12.5) | 5 (7.8) |

|  | **No AP** | **Olanzapine** | **Quetiapine** | **Risperidone** | **Aripiprazole** |
| --- | --- | --- | --- | --- | --- |
| **TOTAL SAMPLE** |  |  |  |  |  |
| *Prolactin, mU/L* | 352.3 (520.1) | 529.7 (418.6) | 348.1 (348.1) | 1254.9 (853.1) | 222.9 (160.9) |
| *Testosterone, nmol/L* | 9.4 (9.8) | 11.0 (9.1) | 8.4 (9.4) | 9.3 (7.7) | 9.9 (9.0) |
| *SHBG, nmol/L* | 48.3 (39.0) | 34.4 (22.7) | 51.4 (45.5) | 39.1 (74.7) | 36.5 (22.3) |
| *Leptin, pmol/L* | 814.3 (817.5) | 835.7 (749.3) | 1143.6 (1091.5) | 913.9 (941.8) | 995.9 (1140.8) |
| *Adiponectin, mg/L* | 12.4 (6.4) | 11.3 (6.1) | 12.3 (6.7) | 11.1 (5.3) | 10.8 (5.6) |
| *Insulin, pmol/L* | 84.5 (59.3) | 100.9 (91.4) | 105.0 (124.1) | 97.8 (74.5) | 89.2 (45.9) |
| *Cortisol, nmol/L* | 426.2 (177.7) | 407.9 (149.2) | 455.5 (166.4) | 417.5 (131.4) | 462.3 (146.4) |
| *TSH, mIE/L* | 2.2 (1.2) | 2.2 (1.2) | 2.4 (1.7) | 2.2 (1.2) | 2.1 (1.3) |
| *fT4, pmol/L* | 14.4 (2.7) | 13.5 (2.4) | 13.2 (2.8) | 14.2 (2.6) | 15.0 (2.8) |
| **MALES** |  |  |  |  |  |
| *Prolactin, mU/L* | 261.6 (173.6) | 381.9 (217.3) | 282.0 (162.6) | 917.8 (462.0) | 161.9 (137.2) |
| *Testosterone, nmol/L* | 18.6 (6.9) | 17.5 (5.8) | 18.0 (6.8) | 14.5 (5.7) | 18.3 (4.6) |
| *SHBG, nmol/L* | 27.4 (11.6) | 25.4 (11.4) | 25.8 (12.6) | 37.6 (97.4) | 23.2 (11.2) |
| *Leptin, pmol/L* | 411.4 (316.4) | 499.9 (378.8) | 486.2 (397.9) | 541.2 (478.9) | 513.3 (397.7) |
| *Adiponectin, mg/L* | 10.2 (5.4) | 9.2 (4.9) | 9.6 (5.2) | 9.1 (4.8) | 8.7 (4.3) |
| *Insulin, pmol/L* | 88.3 (65.9) | 101.9 (69.9) | 114.3 (154.9) | 110.3 (91.3) | 91.5 (45.7) |
| *Cortisol, nmol/L* | 394.1 (142.1) | 389.8 (142.0) | 433.9 (162.7) | 413.4 (125.9) | 467.6 (134.6) |
| *TSH, mIE/L* | 2.3 (1.4) | 2.1 (1.1) | 2.3 (1.2) | 2.2 (1.2) | 2.2 (1.4) |
| *fT4, pmol/L* | 14.8 (2.9) | 13.9 (2.5) | 13.8 (2.7) | 14.4 (2.6) | 15.4 (2.8) |
| **FEMALES** |  |  |  |  |  |
| *Prolactin, mU/L* | 431.1 (683.6) | 746.1 (534.0) | 392.6 (366.0) | 1711.6 (1039.3) | 282.4 (161.5) |
| *Testosterone, nmol/L* | 1.4 (0.6) | 1.4 (0.6) | 1.8 (3.1) | 1.7 (0.7) | 1.6 (0.8) |
| *SHBG, nmol/L* | 66.2 (44.9) | 47.6 (28.1) | 68.6 (51.2) | 41.2 (20.4) | 49.5 (22.9) |
| *Leptin, pmol/L* | 1171.4 (950.0) | 1339.5 (876.7) | 1596.6 (1185.0) | 1454.8 (1170.7) | 1478.7 (1413.8) |
| *Adiponectin, mg/L* | 14.3 (6.6) | 14.4 (6.4) | 14.1 (6.9) | 14.0 (4.6) | 12.9 (6.2) |
| *Insulin, pmol/L* | 81.1 (52.7) | 99.6 (116.7) | 98.6 (97.4) | 79.1 (29.9) | 87.0 (46.7) |
| *Cortisol, nmol/L* | 453.8 (199.7) | 434.8 (155.9) | 470.2 (168.3) | 422.9 (140.4) | 457.1 (158.5) |
| *TSH, mIE/L* | 2.2 (1.2) | 2.3 (1.4) | 2.5 (1.9) | 2.1 (1.3) | 1.9 (1.3) |
| *fT4, pmol/L* | 14.1 (2.5) | 12.9 (2.1) | 12.8 (2.8) | 13.9 (2.7) | 14.4 (2.7) |

**Supplementary Table 2.** Distribution of hormones (unadjusted means (SD)) across antipsychotic (AP) groups, in the total sample and the male/female subsample.

**Supplementary Methods**

**Laboratory analyses of hormones**

The serum levels of the hormones related to the antipsychotic drugs (APs) were analyzed at the Department of Medical Biochemistry, Oslo University Hospital (thyroid hormones) or at the Hormone Laboratory, Oslo University Hospital, Norway (all other hormones). Thyroid-stimulating hormone (TSH) and free thyroxine (fT4) were analyzed on several routine instruments during the period: Architect (Chemiluminesence) from Abbot (2002-2011), Cobas 8000 e602 (2011-2017) and Cobas 8000 e801 (Electrochemiluminescence) (2017-2019) from Roche Diagnostics. Leptin and adiponectin were analyzed with radioimmunoassay (RIA) as specified by the manufacturer, and cortisol and sex hormone-binding globulin (SHBG) were analyzed with Immulite from Siemens during the entire period. Testosterone were analyzed with RIA until 2015, thereafter with an in house Liquid Chromatography Tandem Mass Spectrometry (LC-MS/MS). Insulin was analyzed with RIA until 2015, thereafter by Modular E170 from Roche. Prolactin was analyzed with DELFIA technique until 2017 thereafter with Modular E170 from Roche.

The methodological changes in the laboratory analyses of TSH and fT4 in June 2011 implicated changes in the measured levels. To adjust for the possible confounding effect of these changes we reran the statistical analyses involving the hormones (second hypothesis) by adding the following categorical variable: blood sample taken before June 2011 (N=761) versus blood sample taken after June 2011 (N=413) into the models. The results were similar to the findings found in the main analyses.

For testosterone, insulin and prolactin, we did not adjust for potential changes in measured levels due to the methodological changes, as the numbers of individuals after the methodological changes were low.

**Sensitivity analyses**

We reran the logistic regression analyses in a subsample of patients removing patients with unsatisfactory AP compliance (N=40), and the results were similar to the findings in the main analyses. The results showed a significant main effect for AP groups for increased salivation (No AP vs. aripiprazole, p=0.046), reduced salivation (No AP vs. quetiapine, p=0.003) and nausea/vomiting (No AP vs. olanzapine, p=0.023), but did not reach significant for palpitations (No AP vs. olanzapine, p=0.051) as found in the main analyses. Significant main effect for sex was found for nausea/vomiting (p=0.008), diarrhoea (p=0.025), constipation (p=0.041), polyuria/polydipsia (p=0.008) and palpitations/tachycardia (p=0.026) as in the main analyses. As in the main analyses, a significant interaction effect between sex and AP groups ([males;females x No AP;risperidone] p=0.027) was found for palpitations/tachycardia, and this was associated with prolactin (p=0.010), cortisol (p=0.034) and insulin (p=0.037).
